# Economic evaluation of dialysis and comprehensive conservative care for chronic kidney disease using the ICECAP-O and EQ-5D-5L; a comparison of evaluation instruments

**DOI:** 10.1186/s12962-023-00491-3

**Published:** 2023-11-03

**Authors:** Telma Zahirian Moghadam, Jane Powell, Afshan Sharghi, Hamed Zandian

**Affiliations:** 1 Social Determinants of Health Research Center, Ardabil University of Medical Sciences, Ardabil, Iran; 2 Centre for Public Health and Wellbeing, School of Health and Social Wellbeing, College of Health, Science and Society, University of the West of England, Bristol, UK; 3 Department of Community Medicine, School of Medicine, Ardabil University of Medical Sciences, Ardabil, Iran

**Keywords:** Cost-effectiveness analysis, Dialysis, Comprehensive conservative care, Chronic kidney disease, Quality of life, Elderly

## Abstract

**Background:**

Chronic Kidney Disease (CKD) patients often require long-term care, and while Hemodialysis (HD) is the standard treatment, Comprehensive Conservative Care (CCC) is gaining popularity as an alternative. Economic evaluations comparing their cost-effectiveness are crucial. This study aims to perform a cost-utility analysis comparing HD and CCC using the EQ-5D-5L and ICECAP-O instruments to assessing healthcare interventions in CKD patients.

**Methods:**

This short-term economic evaluation involved 183 participants (105 HD, 76 CCC) and collected data on demographics, comorbidities, laboratory results, treatment costs, and HRQoL measured by ICECAP-O and EQ-5D-5L. Incremental Cost-Effectiveness Ratios (ICERs) and Net Monetary Benefit (NMB) were calculated separately for each instrument, and Probabilistic Sensitivity Analysis (PSA) assessed uncertainty.

**Results:**

CCC demonstrated significantly lower costs (mean difference $8,544.52) compared to HD. Both EQ-5D-5L and ICECAP-O indicated higher Quality-Adjusted Life Years (QALYs) for both groups, but the difference was not statistically significant (p > 0.05). CCC dominated HD in terms of HRQoL measures, with ICERs of -$141,742.67 (EQ-5D-5L) and -$4,272.26 (ICECAP-O). NMB was positive for CCC and negative for HD, highlighting its economic feasibility.

**Conclusion:**

CCC proves a preferable and more cost-effective treatment option than HD for CKD patients aged 65 and above, regardless of the quality-of-life measure used for QALY calculations. Both EQ-5D-5L and ICECAP-O showed similar results in cost-utility analysis.

## Background

Chronic Kidney Disease (CKD) is a pervasive global health challenge, marked by the kidneys' diminished ability to effectively filter waste and fluids from the bloodstream, resulting in the accumulation of toxins and harmful substances. Recent estimates indicate that CKD affects 13.4% of the global population, with prevalence rates ranging from 8 to 16% [1]. In the United States, it is estimated that up to 26 million adults could be affected by CKD. This number has surged over the years, with approximately 10% of the U.S. adult population having CKD between 1988 and 1994, and a significant increase to over 14% in 2019 [2]. Similar trends have been observed globally, with CKD prevalence at 13% in Beijing, China [3], and even higher at 16% in Australia [4]. This substantial rise in CKD rates in the U.S. is likely attributed to the parallel increase in obesity rates and its associated health issues, including diabetes, hypertension, and cardiovascular diseases [2]. This surge in CKD prevalence carries significant economic implications, with associated healthcare costs expected to rise continuously in the foreseeable future [5]. It has also been reported that CKD affects approximately 10% of the global population [6]. This condition is especially concerning in Lower and Middle-Income Countries (LMICs) like Iran, where the cost of Renal Replacement Therapy (RRT) poses a substantial barrier to care access, leading to elevated morbidity and mortality [7].

Of paramount concern is the fact that CKD patients face an elevated risk of cardiovascular disease, which remains the leading cause of early morbidity and mortality among them [8]. Notably, CVD stands as the predominant contributor to both morbidity and mortality in CKD patients. This interplay between CKD and CVD amplifies the public health burden. Epidemiological data underscore the gravity of this association, revealing that a substantial portion of CVD-related fatalities, approximately 7.6%, can be directly linked to impaired kidney function [9]. Such findings underline the urgent need for comprehensive strategies and interventions to address this intricate nexus, reducing the health disparities and heavy economic burden posed by CKD and its interwoven complications in healthcare systems worldwide.

In Iran, there are various treatment options available for CKD patients, including hemodialysis, peritoneal dialysis, kidney transplantation, and Comprehensive Conservative Care (CCC) [10]. Dialysis is the most common form of RRT globally, but it is costly and time-consuming, and the quality of care can differ [11]. CCC, also known as supportive care, is an alternative approach that aims to improve patients' quality of life and symptom management without using RRT [12]. CCC encompasses a wide array of services, including symptom control, non-dialytic imbalance correction, anaemia management, and end-of-life care, all aimed at enhancing patient and family quality of life. For many high-risk patients on CCC, survival rates rival those on dialysis, but the emphasis is on improved quality of life rather than longevity [13].

Observational data suggests that dialysis might not benefit older individuals with multiple comorbidities and poor physical function [14]. Many are now choosing CCC due to challenges with dialysis, emotional toll, and considerations for end-of-life care [15]. CCC offers fewer physical and mental challenges, preserving independence and dignity, and helping patients cope with life-limiting illnesses. The decision hinges on individual values, goals, and health status [15, 16]. Dialysis, while initially helpful for frail elderly, involves extensive hospital visits, longer recovery times, leading many to prefer CCC. Some dialysis patients may see a decline due to comorbidities [17]. However, patients who undergo hemodialysis typically spend almost half of their days attending hospital, which is much higher than the 4% figure for patients who use CCC [18].

Sen's Capability theory, as a popular theory of redistribution, distinguishes between functioning and capability, where capability is the ability to function in a certain way [19, 20]. The ICECAP-O is a capability-based instrument that measures capability well-being through health and non-health dimensions [21, 22]. It allows for the computing of capability, which is different from Health-Related Quality of Life (HRQoL) based on the EQ-5D-5L. The ICECAP-O has been validated and compared to the EQ-5D in various studies, but it is rarely used in economic evaluations, and its properties have not been thoroughly investigated [23–25]. The exploration of these instruments offers a comprehensive perspective, capturing not only traditional health aspects but also the broader notion of capability and well-being. However, it's essential to recognize that each instrument bears its unique strengths and limitations.

The EQ-5D-5L holds paramount importance in our study, especially in Iran, where it is widely used [26], it provides a solid basis for comparing HRQoL outcomes with other studies. Its comprehensive nature allows us to assess healthcare interventions' impact comprehensively, contributing to a broader understanding of HRQoL and cost-effectiveness, both nationally and globally [27]. By including EQ-5D-5L as a comparator, we enhance the robustness and comparability of our study's findings, facilitating informed healthcare decision-making not only in Iran but also internationally. The EQ-5D-5L, while widely used, might not fully capture the holistic impact of CKD on patients' lives. In contrast, the ICECAP-O's capability-based approach is more comprehensive but may require more in-depth data collection. By discussing these aspects, this study aims to provide a thorough understanding of the suitability and implications of employing these tools in assessing CKD treatment outcomes [27]. Furthermore, the utilization of ICECAP-O in cost-effectiveness assessments among older populations is infrequent, as evidenced by a study carried out by Makai et al. [28]. This study focused on frail elderly individuals, aiming to evaluate the cost-effectiveness of an integrated care model within a relatively short time frame. It also sought to explore whether the incorporation of a more comprehensive measure of well-being, based on capabilities, in economic evaluations results in divergent findings regarding cost-effectiveness [28]. ICECAP-O has not been used in cost-effectiveness analyses for elderly CKD patients, which could be a suitable population to assess its usefulness given their diverse health and social care needs.

Existing research on economic evaluations of kidney supportive care, whether for pre-dialysis patients, dialysis patients, or those under conservative management, exhibits notable gaps. These gaps include the need for robust survival estimates across various levels of kidney function and longitudinal assessments of Health-Related Quality of Life (HRQOL), especially among older individuals with multiple health issues, spanning the entire disease trajectory, including end-of-life care [29, 30]. Furthermore, there's a requirement for broader measures of well-being, encompassing capabilities and treatment preferences. Additionally, comprehensive assessments of costs and resource utilization for specialized and community-based kidney supportive care services are lacking [22, 31]. Despite the widespread use of dialysis and Comprehensive Conservative Care (CCC) in high-income countries, there's a shortage of studies comparing the cost-effectiveness of these approaches in Iran. With an ageing population and rising comorbidities among CKD patients receiving kidney replacement therapy, healthcare systems face increasing burdens. Therefore, an economic evaluation of these two approaches is critical for informed decision-making by policymakers and healthcare providers in resource-limited settings.

This paper has two objectives: firstly, to economic evaluation (cost-utility analysis) of HD and CCC with a short-term duration (1-year) for elderly CKD patients, and secondly, to examine whether using a wider measure of well-being (capability) in an economic evaluation (ICECAP-O), in addition to routine measures (EQ-5D-5L), produces a different result in terms of cost-effectiveness.

## Method and materials

### Study design

In this short-run time economic evaluation study, we conducted a cost-utility analysis to compare the costs and HRQoL of two treatment methods, Hemodialysis (HD), and Comprehensive Conservative Care (CCC), for patients with CKD in the northwest region of Iran during November 2021 to May 2022.

### Sampling

The study sample was obtained from the medical records of the Ardabil dialysis centre. Participants were eligible if they met the following criteria:

- Over 65 years old.
- Diagnosed with CKD (people with glomerular filtration rate (GFR) less than 60 ml/min/1.73 m^2^ for three or more months and albumin-to-creatinine ratio (ACR) 30 mg/g or higher (or equivalent protein-to-creatinine ratio [PCR] of 50 mg/mmol or higher).
- Received either HD or CCC as their primary dialysis treatment.
- Medical records (blood tests, urine tests, imaging studies, or kidney biopsy) with complete information on their demographic characteristics, comorbidities, laboratory results, and direct and indirect costs related to their treatment.
- Completed the ICECAP-O and EQ-5D-5L questionnaires to assess their HRQoL at the baseline.

Participants who had received a kidney transplant had cognitive impairments that affected their ability to complete the questionnaires or had other serious medical conditions (such as Dementia) were excluded from the study. There was no matching at baseline, and all patients in each group were above 65 years old.

**Fig. 1:**
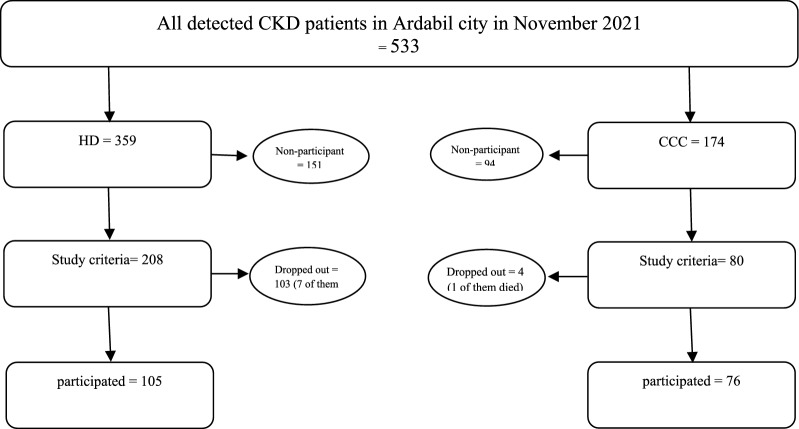
Sampling flowchart

Based on the available data from the Executive summary of the KDIGO Controversies Conference on Supportive Care in Chronic Kidney Disease [32], we assumed the mean difference in Quality-Adjusted Life Years (QALYs) as an indicator of HRQoL between the HD and CCC groups to be 0.03, with no detailed study available on this matter. To calculate the sample size, we assumed a mean difference of 0.03 QALYs and a standard deviation of 0.1, with a two-sided significance level of 0.05 and a power of 0.8. Using the formula:

$${\text{n}}\, = \,({\text{Z}}_{{\left( {\alpha /2} \right)}} \, + \,{\text{Z}}_{\left( \beta \right)} )^{2} \,*\,({\text{SD}}1^{2} \, + \,{\text{SD}}2^{2} )\,/\,({\upmu }_{1} \, - \,{\upmu }_{2} )^{2}$$

where:

- n = sample size per group
- Z_(α/2)_ = critical value of the normal distribution for a significance level of α/2
- Z_(β)_ = critical value of the normal distribution for the power of 1-β
- SD_1_ and SD_2_ = standard deviations of the two groups
- μ1 and μ2 = means of the two groups

we obtained a required sample size of 96 participants (48 in each group). Since our study already has more participants than this calculated sample size, it can be considered adequately powered for the chosen effect size, power, and significance level [33, 34]. Finally, we selected 183 participants, who met the eligibility criteria, to include in the study. Of these, 105 were in the HD group and 76 were in the CCC group (Fig. 1).

### Data collection

Data collection for this economic evaluation took place from November 2021 to May 2022 from two sources, first from the medical records of the Ardabil dialysis centre and second from face-to-face interviews with the patients. Medical-related data, including demographic information, medical history, comorbidities, laboratory results, and information on the duration and frequency of the interventions received, were extracted from the medical documents of all participants before face-to-face interviews, were conducted. Additionally, data on the HRQoL of both groups were collected through face-to-face interviews, which took place between April to May 2022. To accommodate the HD group's schedule, interviews were arranged based on the patient’s preferred time, typically not on the day of receiving dialysis. Conversely, for the CCC group, interviews were scheduled based on the patient’s preferred time on the day of the interview. The interview began with two general questions, namely the participants' age and the time of their last visit to a doctor's office or hospital, to mentally prepare them. Next, the participants were asked to choose one of two envelopes, each containing the name of a questionnaire. The participants were then given the selected questionnaire to answer. To minimize any potential bias, the participants were asked another general question about their family (the names of their children and grandchildren) after completing the first questionnaire. Finally, the participants were asked to complete the second questionnaire.

To ensure data quality, all data were collected by trained research assistants who underwent a rigorous training program before starting data collection. Data were also double-checked for accuracy and completeness by a separate team of data managers. Any discrepancies or missing data were resolved by contacting the patient or referring physician. In our study, it was observed that a significant proportion of participants, particularly in the CCC group, had varying levels of literacy challenges. To ensure the accuracy and reliability of data collection, we implemented a rigorous approach. Interviewers involved in data collection underwent comprehensive training to administer the quality-of-life instruments consistently and sensitively. For participants with literacy difficulties, interviewers read the questions aloud and recorded responses verbatim to minimize reporting bias. Furthermore, we incorporated sensitivity analyses in our study design to assess the potential impact of literacy-related factors on our findings. This approach not only allowed us to address potential biases but also ensured that our study maintained a high standard of data quality and integrity.

All data were stored in a secure, password-protected database with limited access. Data were regularly backed up to prevent loss or corruption, and the database was regularly maintained and updated to ensure data accuracy and completeness.

This expanded version of the data collection section provides more details on how cost and HRQoL data were collected, and other data collected in the study. It also includes information on data quality assurance and management, which are important aspects of ensuring the validity and reliability of study findings.

### Cost data

**Fig. 2:**
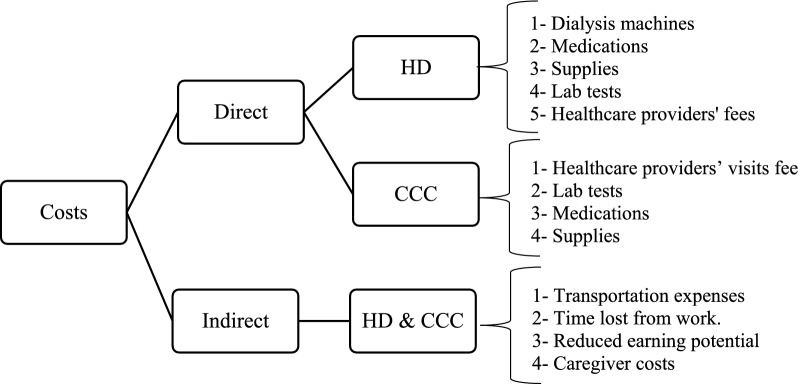
Breakdown of direct and indirect costs for HD and CCC patients

We collected cost data using a detailed approach, ensuring we accounted for all expenses linked to each treatment method. This included direct medical costs, like those found in hospital records, such as hospitalization, medications, lab tests, and doctor visits. We also considered indirect costs, like those associated with transportation and time. Indirect costs were estimated by interviewing patients and calculating the impact of missed work and lost income. For patients receiving HD, direct costs encompassed dialysis machines, medications, supplies, lab tests, and healthcare providers' fees. Conversely, for those in the CCC group, direct costs involved visits to various healthcare providers, lab tests, medications, and necessary supplies. Indirect costs for both groups included transportation expenses, time lost from work, reduced earning potential, and caregiver costs. We aimed to provide a comprehensive view of all financial aspects related to these treatments (Fig. 2).

All costs were measured in Iranian Rials (IRR) and were converted to US dollars (USD) using the official exchange rate at the time of data collection.

### Validity and applicability of economic assessment tools: EQ-5D-5L and ICECAP-O

The validity and applicability of economic assessment tools, such as EQ-5D-5L and ICECAP-O, are critical considerations in health economic studies. In this context, EQ-5D-5L is a widely recognized and extensively validated instrument for assessing HRQoL [23–28, 35]. It provides a comprehensive overview of an individual's health status across multiple dimensions, including mobility, self-care, usual activities, pain/discomfort, and anxiety/depression. The EQ-5D-5L has been validated in various populations and can generate utility scores that can be used in economic evaluations. Its applicability lies in its ability to provide a standardized measure of HRQoL that can be compared across different health interventions and settings.

On the other hand, ICECAP-O is a capability-based instrument designed to capture the well-being and capabilities of older individuals. While it lacks a preference-based scoring system for directly calculating Quality-Adjusted Life Years (QALYs), it offers a valuable perspective on broader aspects of quality of life that are particularly relevant to older populations. ICECAP-O focuses on attributes like attachment, security, role, enjoyment, and control, providing a more holistic view of well-being. Its applicability is significant in studies where the goal is to capture a more comprehensive picture of the impact of healthcare interventions on individuals' lives, especially in scenarios involving older or frail populations.

Both instruments, EQ-5D-5L and ICECAP-O, offer unique strengths and can be chosen based on the specific research objectives and the population under study. The validity of these instruments largely depends on their appropriate adaptation and validation in the target population. Ensuring that these tools are culturally relevant and sensitive to the population's characteristics is crucial for obtaining meaningful and applicable results in economic evaluations. Moreover, it's essential to transparently report the methods used to derive utility scores or QALYs from these instruments, as this can significantly impact the results and their applicability in healthcare decision-making [36].

### Outcomes and covariates

#### Health-Related Quality of Life (HRQoL) & Quality-Adjusted Life Years (QALYs)

This study places significant importance on HRQoL as a crucial indicator of health outcomes and as the primary outcome measure. To assess HRQoL, QALYs were calculated for each patient using the ICECAP-O and EQ-5D-5L questionnaires administered in May 2022. QALYs are a measure of health outcomes that combines quantity and quality of life. They are commonly used in cost-effectiveness analyses to compare the value of different healthcare interventions [37]. The EQ-5D-5L is a generic instrument that measures health-related quality of life across five dimensions: mobility, self-care, usual activities, pain/discomfort, and anxiety/depression. The EQ-5D-5L health states transformed into a “utility” score by utilizing a scoring algorithm based on public preferences. Additionally, the EQ-5D-5L instrument features a Visual Analogue Scale (EQ-VAS) that is used to obtain a global rating of self-perceived health. The EQ-VAS is measured on a 0–100 mm scale, where 0 represents the worst possible health state and 100 represents the best health state imaginable [24, 38].

ICECAP-O is a measure of capability well-being that focuses on the attributes that are important to older people. It includes five domains: attachment, security, role, enjoyment, and control. Each domain has four response options, ranging from “no capability” to “full capability” [24, 39]. ICECAP-O is a valuable measure of the quality of life in older adults, but it does not have a preference-based scoring system to derive QALYs, so we passed a few steps to calculating QALYs based on the ICECAP-O.

### Data analysis

Descriptive statistics were calculated to summarize the characteristics of the study sample, including age, sex, duration of CKD, comorbidities, and laboratory results. Means and standard deviations were used for continuous variables, and frequencies and percentages were used for categorical variables. In addition, statistical tests were used to compare between two groups in terms of baseline characteristics.

Direct and indirect costs were analyzed separately. The total cost per patient for each intervention was calculated by summing the costs of all resources used by patients to use the intervention.

To calculate HRQoL using QALYs, the health utility score obtained from the instruments (EQ-5D-5L and ICECAP-O) is multiplied by the time spent in a particular health state to get the number of QALYs gained or lost. The literature was consulted to obtain these quality-of-life estimates, which range from zero (representing death) to one (representing full health) [40, 41]. For example, if a patient has a health utility score of 0.7 while in a certain health state for two years, the number of QALYs gained or lost would be 1.4 QALYs (0.7 × 2 years). In this study, we defined one year as a time horizon based on previous studies [42]. To calculate QALYs based on ICECAP-O, we first assigned a score to each response option for each domain. The scores range from 0 to 1, with 1 representing full capability and 0 representing no capability. Once we assigned scores to each response option, we calculated the total ICECAP-O score for everyone by summing the scores across all five domains. The maximum possible score is 5. In the next step, we converted the total ICECAP-O score into a utility score using a mapping function^1^ extracted from previous studies in Iran [43]. The mapping function translates the ICECAP-O score into a utility score, which ranges from 0 to 1, with 1 representing perfect health and 0 representing death. Finally, we calculated the QALYs by multiplying the utility score by the time spent in a particular health state. For example, if an individual spends one year in a health state with an ICECAP-O utility score of 0.5, the QALYs gained would be 0.5 [35].

In this study, we extracted the Iranian population norms for the EQ-5D-5L questionnaire from the literature [43] to calculate the utility values for our study population. All expenses were reported in 2021 and adjusted to present value using a local discount rate of 6.0% [12, 44] for both costs and outcomes. The country's threshold for cost-effectiveness was determined to be three times its GDP per capita, which is approximately 520 million IRR ($12,380) per QALY. The costs were estimated using the 2022 US dollar exchange rate [45]. The patient’s health status was evaluated using the Euro QoL EQ-5D-5L Persian version [43], with participants being asked about their current and past states of health.

We did not find an appropriate method to combine both instruments in each other to calculate QALYs, so our approach was to calculate QALY based on both instruments separately. This approach enabled us to compare the net results of instruments in QALYs calculation and extract both health-related and broader aspects of quality of life [21].

### Incremental cost-effectiveness ratio (ICER)

The Incremental Cost-Effectiveness Ratio (ICER) is a metric used to compare the cost difference between two treatments with their respective outcomes, usually measured in Quality-Adjusted Life Years (QALYs) gained [46].

To calculate ICER, we first find the cost difference between the two treatments (HD and CCC) by subtracting the total cost of the less expensive treatment from the total cost of the more expensive one. Then, we calculate the QALY difference between the two treatment groups for each instrument separately by subtracting the total QALYs gained in the less effective treatment group from that in the more effective group. Finally, we divide the cost difference by the QALY difference to obtain the ICER. This value represents the additional cost per additional QALY gained for the more expensive treatment compared to the less expensive one. The formula used for ICER calculation is as follows:

$${\varvec{ICER}}=\frac{({\varvec{Cost}}\,\boldsymbol{ }{\varvec{of}}\,\boldsymbol{ }{\varvec{HD}}-{\varvec{Cost}}\,\boldsymbol{ }{\varvec{of}}\,\boldsymbol{ }{\varvec{CCC}})}{({\varvec{QALYs}}\,\boldsymbol{ }{\varvec{gained}}\,\boldsymbol{ }{\varvec{with}}\,\boldsymbol{ }{\varvec{HD}}-{\varvec{QALYS}}\,\boldsymbol{ }{\varvec{gained}}\,\boldsymbol{ }{\varvec{with}}\,\boldsymbol{ }{\varvec{CCC}})}$$

where:

- Cost of HD = total cost of providing hemodialysis treatment to the patients in the HD group
- Cost of CCC = total cost of providing comprehensive conservative care to the patients in the CCC group
- QALYs gained with HD = total QALYs gained by the patients in the HD group during the study period
- QALYs gained with CCC = total QALYs gained by the patients in the CCC group during the study period

In this evaluation, the cost-utility threshold (willingness to pay (WTP)) was considered equal to Iran's one-time GDP per capita in 2022, equivalent to 25,249 Dollars (70 million Rials) [47, 48]. Rial values in the present study were converted using the purchasing power parity (PPP) Dollar conversion factor to Rial equal to 42,157 Rials (Average exact exchange rate in 2021: 421,570.0935 IRR, where we removed a zero to better calculate the costs) [45, 49].

We used Net Monetary Benefit (NMB) to detect the difference between the monetary value of total expected QALYs and total expected costs. To calculate the NMB, we used the following formula:

$${\text{NMB}}\, = \,\left( {{\text{WTP}}\, \times \,{\text{QALY}}} \right)\, - \,{\text{Cost}}$$

where WTP is the willingness-to-pay threshold (in this study, $25,249), QALY is the quality-adjusted life year gained, and Cost is the cost of the intervention.

### Sensitivity analysis

Sensitivity Analysis is a critical component of this study, aiming to account for uncertainties inherent in various model parameters. We conducted a Probabilistic Sensitivity Analysis (PSA) to comprehensively assess how these uncertainties might impact our results. PSA was executed through Monte Carlo simulations, a powerful technique that allows for the integration of various uncertain variables [50]. Our probabilistic model encompassed key input parameters, including the costs associated with Hemodialysis (HD) and Comprehensive Conservative Care (CCC), utility scores derived from both the ICECAP-O and EQ-5D-5L instruments and mortality rates. The probability distributions for these input parameters were meticulously determined. These distributions were informed by a synthesis of our study's data, extensive literature reviews, and insights from expert opinions. This approach ensured that we considered a range of potential scenarios and uncertainties, accommodating variations in the values of these critical parameters. By subjecting our economic evaluation to this rigorous sensitivity analysis, we aimed to provide a more comprehensive and robust assessment of the economic implications associated with HD and CCC for individuals with CKD. This process enhances the credibility of our findings and contributes to a more informed decision-making process for healthcare policymakers and providers in resource-limited settings.

All analyses were performed using standard statistical software (STATA ver. 17) and Excel, and statistical significance was set at p < 0.05.

## Results

A total of 181 participants were included in the study, of which 105 were in the hemodialysis (HD) group and 76 were in the Comprehensive Conservative Care (CCC) group. The two groups were comparable in terms of age (t = -0.348, p = 0.285), gender (χ^2^ = 3.734, p = 0.053), education (χ^2^ = 2.927, p = 0.405), marital status (χ^2^ = 2.096, p = 0.554), smoking (χ^2^ = 0.987, p = 0.302) and comorbidities at baseline (Table 1), where there was no significant difference in two groups in baseline characteristic.

**Table 1:** Baseline characteristics of the study participants

Variable	HD group (n = 105)	CCC group (n = 76)	P-value
Age (years), mean ± SD	76.5 ± 4.33	77.07 ± 7.9	0.728
Gender, n (%)			
Male	61 (58.1)	34 (44.7)	0.053
Female	44 (41.9)	42 (55.3)	
Education, n (%)			
Illiterate	52 (49.2)	28 (37.1)	0.405
Primary	28 (27.1)	20 (26.9)	
Secondary	21 (19.8)	19 (24.8)	
Academic	4 (3.9)	9 (11.2)	
Marital status, n (%)			
Never married	8 (7.4)	2 (3.2)	0.554
Married	85 (81.2)	64 (84.5)	
Widowed	10 (9.2)	6 (8.1)	
Divorced/Separated	2 (2.2)	3 (4.2)	
Smoking status, n (%)			
Yes	30 (28.6)	24 (32.1)	0.320
No	75 (71.4)	52 (67.9)	
Comorbidities, n (%)			
Diabetes	70 (66.7)	49 (64.5)	0.759
Hypertension	85 (81.0)	60 (78.9)	0.739
Coronary artery disease	24 (22.9)	19 (25.0)	0.738
Cerebrovascular disease	15 (14.3)	10 (13.2)	0.828
Peripheral vascular disease	10 (9.5)	7 (9.2)	0.943

**Fig. 3:**
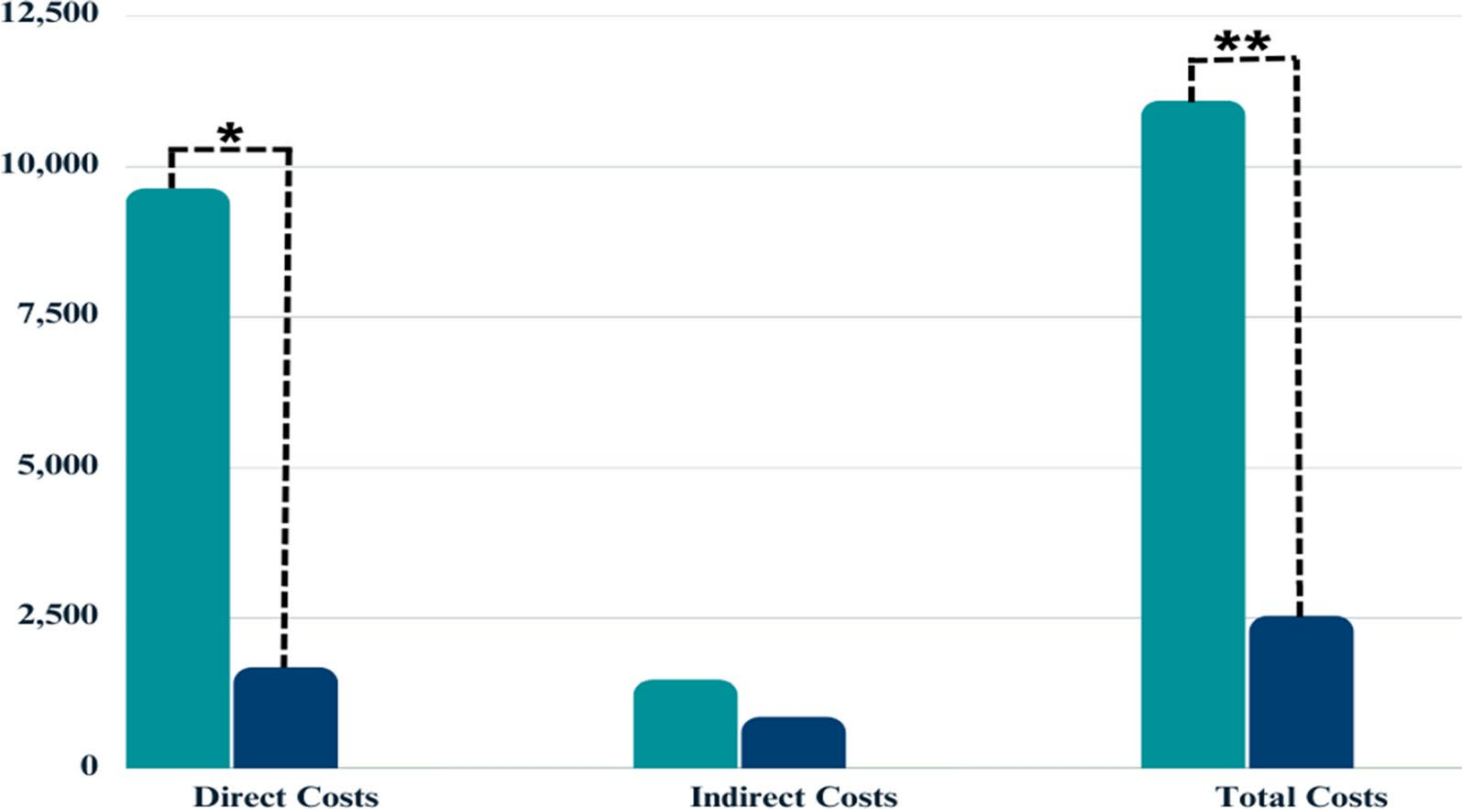
Direct, indirect, and total costs of HD and CCC, indicating a statistically significant difference between the two in terms of direct (*) and total (**) costs

Figure 3 shows the direct, indirect, and total costs of two interventions, where cost was also significantly lower for the CCC group ($2,540.48 ± $306.3) compared to the HD group ($11,085.01 ± $2,188). Direct costs for HD and CCC were $9,621.78 ± $1,899.8 and $1,686.65 ± $61.73, respectively. There was significant difference between two groups in terms of direct costs (p < 0.001). Indirect costs were estimated to be $1,463.22 ± $288.9 for the HD group and $853.82 ± $298.9 for the CCC group, but there was no significant difference between two groups in terms of indirect costs.

**Fig. 4:**
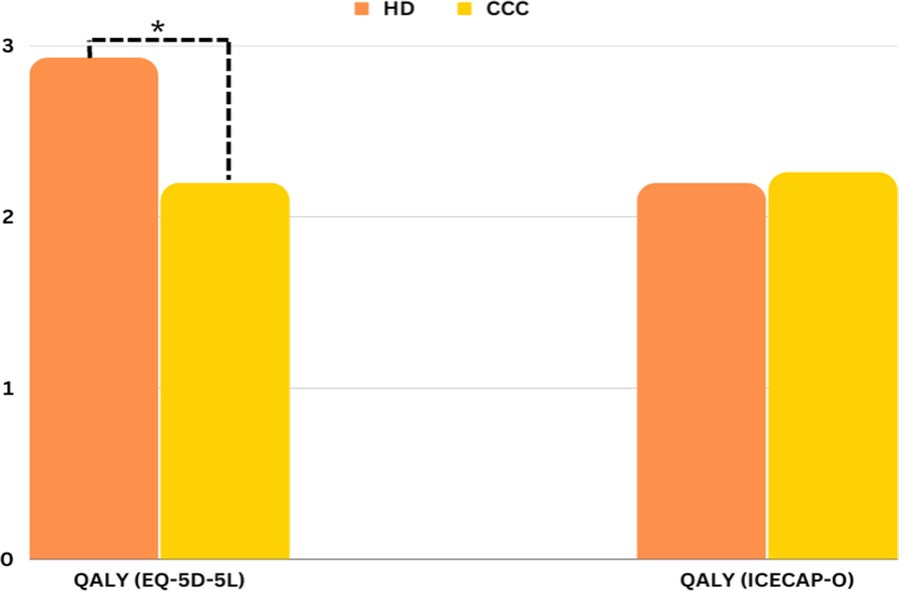
Comparison between HD and CCC in terms of QALY based on two different instruments, indicating a statistically significant difference between the two groups in terms of QALY (*) based on EQ-5D-5L

In Fig. 4, we present a comparative analysis of HRQoL in patients undergoing HD and those receiving CCC. Two distinct instruments, EQ-5D-5L and ICECAP-O, were employed to assess QALYs. Notably, our analysis reveals a statistically significant difference in QALY between the two groups based on EQ-5D-5L (*). This finding underscores the importance of selecting the appropriate instrument for HRQoL evaluation in CKD patients.

Table 2 presents the results of the cost-utility analysis. The mean total cost for CCC was significantly lower than HD, with a difference of $8,544.52. The results showed that the QALYs for both HD and CCC groups were higher when calculated using ICECAP-O compared to EQ-5D-5L. Specifically, the QALYs for HD and CCC using ICECAP-O were 2.93 ± 1.25 and 2.94 ± 1.21, respectively. In contrast, the QALYs for HD and CCC using EQ-5D-5L were 2.19 ± 0.94 and 2.25 ± 0.96, respectively. The difference in QALYs between the two groups was not statistically significant for either ICECAP-O or EQ-5D-5L. When we compared the incremental cost-effectiveness ratios (ICERs) between HD and CCC, we found that CCC was dominant over HD using both HRQoL measures. The ICER based on EQ-5D-5L was -$141,742.67 and based on ICECAP-O was -$4,272.26, indicating that CCC was both less costly and more effective than HD. Therefore, the ICER indicates that for every additional QALY gained from using HD instead of CCC, an extra cost of $427,226 is incurred. These results suggest that CCC is a more cost-utility treatment option compared to HD for patients with CKD who are 65 years and older, regardless of the HRQoL measure used to calculate QALYs.

**Table 2:** Results of the cost-utility analysis

Outcome	HD group	CCC group	Difference
Total cost (USD), mean ± SD	$11,085 ± $2,188	$2,540.48 ± $306.26	$8,544.52
QALYs _based on EQ-5D-5L_	2.19 ± 0.94	2.25 ± 0.96	−0.06
QALYs _based on ICECAP-O_	2.93 ± 1.25	2.94 ± 1.21	0.02
ICER (EQ-5D-5L)	−$141,742.67		CCC is dominant
ICER (ICECAP-O)	−$4,272.26		CCC is dominant
Net Monetary Benefit (NMB) _based on EQ-5D-5L_	−$311,919.01	$28,276.17	−$283,642.84
Net Monetary Benefit (NMB) _based on ICECAP-O_	$44,530.84	$44,110.67	$420.17

NMB was calculated for both the HD group and the CCC group based on WTP threshold of Iran's one-time GDP per capita in 2022 [47, 48]. The results showed that the NMB was positive for the CCC group, indicating that this intervention was economically feasible. On the other hand, the NMB for the HD group was negative, indicating that the costs of this intervention outweighed its benefits. Therefore, it can be concluded that the CCC intervention is the preferred option based on its economic feasibility. These findings highlight the importance of considering both the clinical outcomes and economic consequences of healthcare interventions when making decisions about resource allocation.

### Sensitivity analysis

**Fig. 5:**
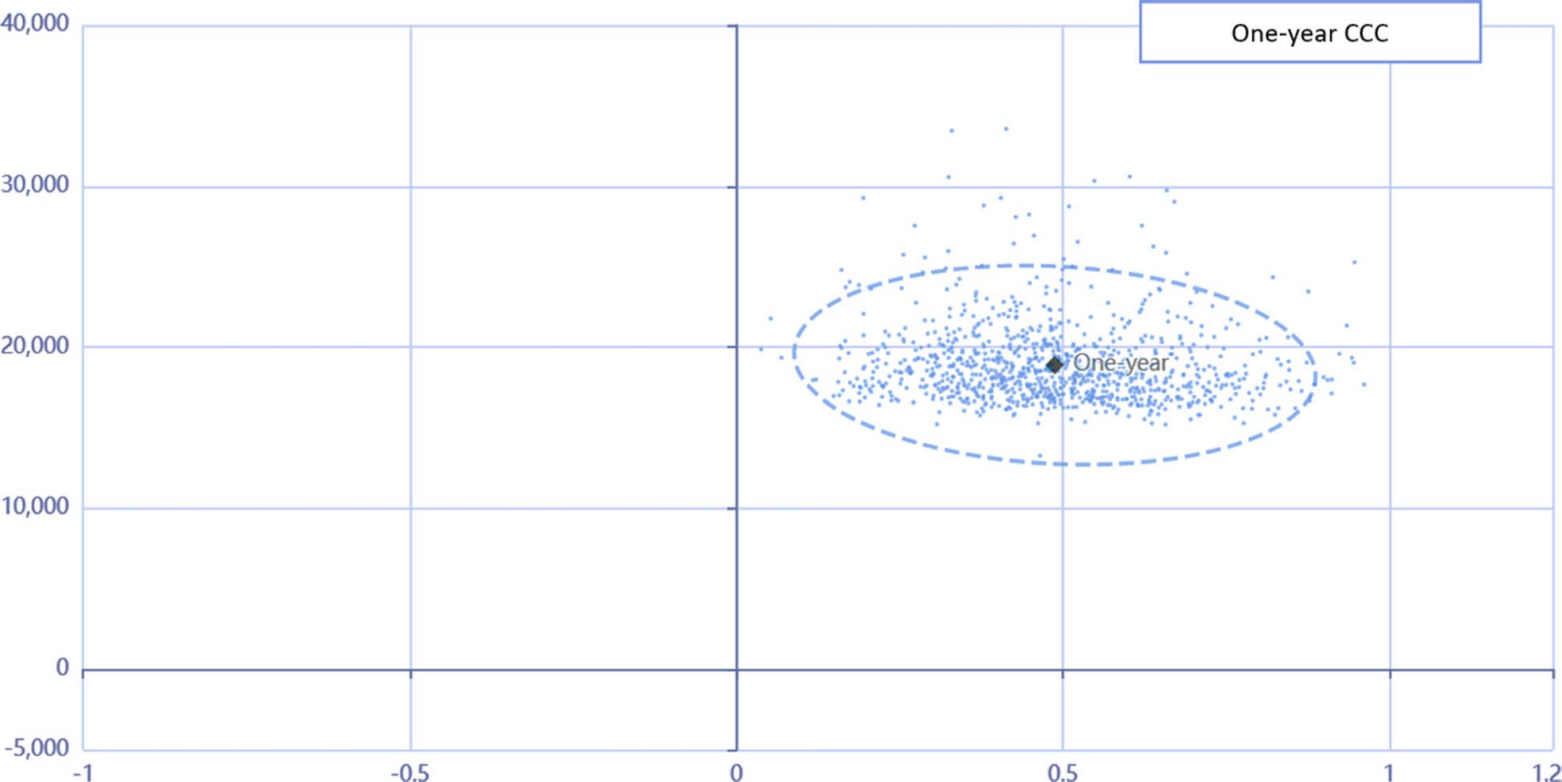
Scatter plot for 1000 results of PSA for HD and CCC. The ellipses represent 95% confidence intervals

To further explore the robustness of our findings (Table 3), we conducted a probabilistic sensitivity analysis (PSA) using a simulation model. The input parameters of the model were assigned probability distributions based on the available data and expert opinion. A Monte Carlo simulation was then performed to generate a range of possible outcomes based on these distributions. A 6% discount rate for both costs and QALYs in the model was used based on the Health Technology Assessment Office of Iran's Ministry of Health recommendation. We generated 1,000 random iterations for each input parameter and calculated the Net Monetary Benefit (NMB) for both HD and CCC treatments at Iran's one times the GDP per capita in 2022, equivalent to 25,249 dollars (Fig. 5). The results of PSA showed that CCC remained the dominant treatment option in most iterations. Specifically, out of 1,000 iterations, CCC was cost-effective in 921 iterations (92.1%), whereas HD was cost-effective in only 79 iterations (7.9%). The results of our PSA suggest that the conclusion drawn from our base-case analysis is robust and that CCC is a more cost-effective option compared to HD for patients with CKD who are 65 years and older.

**Table 3:** Results of probabilistic sensitivity analysis for cost-utility analysis of hemodialysis (HD) and comprehensive conservative care (CCC) for patients with chronic kidney disease (CKD)

	Mean	S. D	Median	Baseline	L.cost	H.cost	Prob
CCC	$70,557.71	$29,582.7	$70,846.14	$25,249	$1,987	$3,095	92.1%
HD	$63,788.18	$30,554.43	$64,061.02		$7,147	$14,244	7.9%

*S.D* Standard Deviation, *L.cost* Low costs, *H.cost* High costs, *Prob* probability of being cost-effective

However, the PSA also identified several input parameters that had a significant impact on the results. These included the cost of the intervention, the discount rate, and the probability of adverse events. Further sensitivity analyses were conducted to evaluate the impact of these parameters on the results, and it was found that the results were sensitive to changes in these parameters. Overall, the results of the PSA suggest that the intervention is likely to be cost-effective, but the results are sensitive to certain input parameters. Therefore, further research and data collection are needed to reduce the uncertainty in these parameters and to increase the robustness of the results [51].

## Discussion

The study compared the cost-effectiveness of Hemodialysis (HD) versus Comprehensive Conservative Care (CCC) for patients with CKD using two different HRQoL measures: the EQ-5D-5L and ICECAP-O instruments. Previous studies on CKD or End Stage Renal Diseases (ESRD) patients have mainly focused on examining patient survival rates [52–54] or cost-effective analysis of various dialysis methods, such as home dialysis, peritoneal dialysis, or hemodialysis [55]. Many studies that have examined and compared different treatment methods for CKD patients in terms of HRQoL have been conducted in developed countries [56, 57] where CCC is more advanced compared to developing countries like Iran. Morton et al. reviewed the literature and identified gaps in the evidence and challenges associated with measuring the costs, benefits, and cost-effectiveness of kidney-supportive care. They emphasized on economic evaluation of CCC compared to other treatment methods for patients with chronic kidney disease [30]. In addition, there has been a lack of studies that have simultaneously used two health-related quality-of-life measurement tools, where most of them just used one version of instruments [58] or an old version [59]. Studies that have focused on HRQoL in two groups of patients undergoing HD and CCC have also been limited to ESRD patients and focused on developed countries such as England [60, 61]. In this study, we used two different HRQoL and capability measurement tools, EQ-5D-5L and ICECAP-O, and calculated QALY based on them separately.

A total of 181 participants were included in the study, with 105 in the HD group and 76 in the CCC group. The prevalence of CCC is lower than HD due to the lack of awareness and training among the public and healthcare providers and its lower survival rate. Jong et al. classified the hindrances to implementing CCC in CKD patients into three categories: patient-related factors such as attitude, motivation, knowledge, socio-cultural background, and role perception, healthcare professional-related factors including communication skills, working style, and fears and concerns, and healthcare system-related factors like financial constraints, practice organization, and availability of supportive staff [62]. A systematic review revealed that the approach of CCC is centred on symptom management and holistic care, rather than invasive interventions like HD and elderly patients with CKD who undergo CCC experience a maintained quality of life (QOL) but with a lower survival rate compared to those who opt for dialysis treatment [63]. When deciding on the treatment option, the patient's preferences, and goals for their QOL should be taken into consideration. CCC as an additional form of support can help healthcare providers, patients, and caregivers make an informed decision and implement the most appropriate treatment approach that aligns with the patient's healthcare objectives [60].

The study found that the two groups were comparable in terms of baseline characteristics, including age, gender, education, marital status, smoking, and comorbidities. Several studies showed that there are no differences between patients managed conservatively and dialysis patients on baseline characteristics, physical, and mental health summary scores [61]. For instance, Verbeme et al. demonstrated in their research that there were no variations in the comorbidity score, primary diagnosis of kidney disease, body mass index, serum albumin, or C-reactive protein level between the two HD and CCC groups [61].

Moreover, there are several reasons underlying the observed differences in healthcare resource utilization between the CCC and HD groups that investigate the substantial disparity in healthcare resource utilization between the CCC and HD groups reveals a complex interplay of factors that contribute to the reduced treatment burden observed in the CCC cohort. Firstly, the patient-centred nature of CCC, which places a strong emphasis on holistic care and symptom management, inherently reduces the need for frequent outpatient visits [64]. This proactive approach to addressing patients' needs within the community setting not only ensures better symptom control but also minimizes the necessity for routine medical check-ins. Secondly, the disparity in hospitalization rates between the two groups warrants scrutiny. Patients undergoing HD often contend with a higher risk of complications related to the procedure itself, including infections and vascular access issues, which can culminate in hospital admissions [65, 66]. Conversely, CCC's emphasis on patient education and self-management equips individuals with the tools to manage their health conditions effectively, potentially mitigating the need for hospital stays [18, 57]. Moreover, the CCC model's proactive approach to managing health issues in an outpatient setting likely results in timely interventions, preventing the exacerbation of health problems that might otherwise necessitate hospitalization. Collectively, these factors translate into a higher number of hospital-free days for CCC patients, signifying not only improved patient well-being but also potential cost savings within the healthcare system [16, 57, 67].

Based on our results, the cost of the intervention was significantly lower for the CCC group compared to the HD group from the patient’s perspective, with direct costs for HD and CCC being $9,621.78 ± $1,899.8 and $1,686.65 ± $61.73, respectively. The indirect costs were also lower for CCC, although there was no significant difference between the two groups in terms of indirect costs. The level of treatment burden is a significant concern for kidney patients, especially when deciding between HD and CCC, and the used perspective to calculate the burden [52–54, 68]. Despite this, there are only a limited number of studies that have compared the treatment burden between the two options. Furthermore, some of these studies lack detailed definitions and do not specify which aspects of treatment burden were assessed, such as in-centre hemodialysis days or outpatient visits. Patients undergoing dialysis tend to experience higher hospitalization rates and spend more time in the hospital compared to those receiving CCC treatment [67, 69, 70]. Previous evidence, which was conducted from health provider perspective, showed that CCC had a significantly reduced treatment burden when compared to those receiving HD. This was evident through fewer outpatient visits, hospital admissions, and in-hospital days, ultimately leading to a higher number of days without hospitalization [61].

Regarding the HRQoL measures, the study found that both HD and CCC groups had higher QALYs when calculated using ICECAP-O compared to EQ-5D-5L. However, the two groups had a statistically significant difference in QALYs based on EQ-5D-5L, with CCC having a higher QALY score. The EQ-5D-5L is a commonly used method to evaluate health-related quality of life in patients with various illnesses across the globe. The utility values for the same illness differ depending on the patients' attributes, living environment, and the value set of EQ-5D-5L [71, 72]. This study showed that the mean EQ-5D VAS scores were 67.32 (± 6.42) and 75.15 (± 11.17) for HD and CCC, respectively. The health utility values for CKD patients varied in different studies [67, 71, 72]. The highest and lowest values for VAS in patients with CKD were reported from Japan and the UK, respectively [71, 73]. Patients who had been on dialysis for 4 years or longer reported the lowest value of VAS and self-care was identified as the dimension with the most reported problems among CKD patients [67, 71, 73]. The meta-analytic utility estimate in a systematic review revealed that CKD patients have lower HRQoL score in compare with other patients [74] which is in line with this study’s results.

Several studies showed that in elderly patients with CKD, both CCC and HD have similar patient outcomes. However, in patients with end-stage renal disease (ESRD), CCC is associated with shorter survival and increased mortality compared to HD. This difference was not observed in patients with severe comorbidities. In addition, scientific literature demonstrated that quality of life, symptom prevalence, and functional outcomes were similar in patients managed conservatively and those receiving dialysis care for CKD. For instance, Ren et al. [75] showed in their study that approximately 55% of patients undergoing CCC experienced stable or improved quality of life and symptom relief in prospective cohort studies. However, there were no significant differences in quality of life and symptoms between CCC and renal replacement therapy in their study [75]. Busa et al. [76]. In a recent investigation by Busa and colleagues (2022), they sought to evaluate the quality of life in older individuals with chronic kidney disease by comparing the utility scores obtained from both ICECAP-A and EQ-5D-5L. Their analysis revealed that there were no statistically significant differences in the mean utility scores among different subgroups for EQ-5D-5L or ICECAP-A [76]. These results align with the findings presented in our study. In our study patients managed with HD reported a higher burden of kidney disease compared to patients managed with CCC, but the overall quality of life was not significantly different between the two groups.

There is limited evidence of comparison between CCC and HD in terms of cost-utility analysis and from the patients’ perspective, where most of the studies were conducted to compare the cost-effectiveness of different dialysis methods from the perspective of the service provider and on patients with ESRD [77–79]. The results of the cost-utility analysis in this study from the patients’ perspective showed that CCC was dominant over HD using both HRQoL measures, with an ICER indicating that for every additional QALY gained from using HD instead of CCC, an extra cost of $427,226 is incurred, this means that the CCC intervention produces better outcomes than the HD intervention. We calculated NMB for both the HD and CCC groups based on a WTP threshold of Iran's one-time GDP, where the results revealed a positive NMB for the CCC group, indicating that this intervention was economically feasible. Conversely, the NMB for the HD group was negative, which implies that the costs of this intervention outweighed its benefits. The economic evaluation results suggest that the CCC intervention is the preferred option based on its economic feasibility. Overall, the study found that CCC was a more cost-effective intervention than HD for older patients with CKD and that the choice of HRQoL measure did not have a significant impact on the cost-effectiveness analysis results. A study conducted in Indonesia revealed that, at a willingness-to-pay threshold of 43 million Indonesian Rupiah, supportive care was identified as the most cost-effective treatment option [79] which is in line with this study's findings. Smedt et al. conducted a study in Belgium to compare the cost-effectiveness of continuous renal replacement therapy with intermittent renal replacement therapy and conservative care treatment for acute kidney injury. The study found that while renal replacement therapy was more expensive than conservative treatment, it did not result in a significant increase in QALYs. Therefore, they concluded that from a health economic standpoint, conservative treatment seems to be the preferred treatment strategy [80]. In contrast, there are some studies that ignore our findings such as a study in Canada showed that regarding the cost-effectiveness of CCC was limited, low to moderate quality and not generalizable to all settings [81] or a study that demonstrated that the dialysis group lived approximately twice as long compared to the CCC group, but CCC patients scored significantly lower on Physical Component Summary and Mental Component Summary, symptoms, and effects of kidney disease on daily life, while no differences were observed on burden of kidney disease [61].

Finally, it could be concluded that CCC can be a cost-effective treatment option for CKD patients, especially from the patient's perspective. The cost of intervention for CCC was significantly lower than HD from the patient's perspective. The level of treatment burden is a significant concern for kidney patients, especially when deciding between HD and CCC. In addition, CCC had a significantly reduced treatment burden when compared to those receiving HD, which was evident through fewer outpatient visits, hospital admissions, and in-hospital days, ultimately leading to a higher number of days without hospitalization. When deciding on the treatment option, the patient's preferences, and goals for their QOL should be taken into consideration. Thus, different HRQoL measures can lead to different conclusions regarding the effectiveness of different treatments, and both should be considered when making healthcare decisions.

Based on the study's findings, there are several areas for future research. Firstly, there is a need for further investigation into the cost-effectiveness of CCC compared to other treatment options for CKD patients, particularly in developing countries where CCC may be less advanced. Secondly, future studies should focus on comparing the treatment burden between CCC and HD, including outpatient visits, hospital admissions, and in-hospital days. Additionally, there is a need for more studies that simultaneously use multiple HRQoL measurement tools, as this can provide a more comprehensive understanding of the impact of treatment on patients' quality of life. Finally, future research should explore the patient-related, healthcare professional-related, and healthcare system-related factors that hinder the implementation of CCC in CKD patients, and strategies to overcome these barriers to improve access to this treatment option.

One of the significant strengths of this study is that it is the first of its kind to use two different instruments to calculate QALYs for cost-utility analysis. The use of both the ICECAP-O and EQ-5D-5L in the study helps in providing a more comprehensive evaluation of the quality of life of patients receiving CCC and HD. Additionally, this study is also the first cost-utility analysis of CCC and HD in Iran, which is a developing country. This factor adds to the significance of the study's findings, particularly considering the lack of research in RRT cost-utility analyses in developing countries. Furthermore, this study used a patient perspective for the cost-utility analysis of two interventions for comparison purposes. This approach provides a more holistic and complete picture of the overall impact of CCC and HD on patients' lives, particularly in terms of quality of life.

One of the limitations of this study is that it was a retrospective study and, therefore, may be subject to potential bias and confounding, particularly due to the lack of randomization and the potential for missing data. The authors attempted to address these limitations by carefully selecting study participants and using appropriate statistical methods. However, the retrospective nature of the study still poses a risk of confounding and bias. Another limitation is that the study calculated QALY based on ICECAP-O using EQ-5D-5L utility score because no previous study had calculated a utility score for ICECAP-O. While this approach provides valuable insights, it also poses a risk of inaccuracies in the QALY calculations. One notable limitation of this study was the utilization of a one-off interview approach to assess patients' health-related quality of life (HRQoL). While repeated assessments over time can provide a more comprehensive understanding of HRQoL dynamics, we chose a single interview for several reasons. Finally, the study did not consider any evidence on repatriation, frailty, renal function, and safety outcomes when comparing CCC and HD, which may have provided a more complete picture of the differences between the two interventions. Finally, the study was conducted in northwest Iran, and its generalizability to other contexts should be considered.

## Conclusion

In conclusion, this study highlights the cost-effectiveness of CCC compared to HD in patients over 65 with CKD, as determined by their HRQoL. The findings suggest that CCC may provide greater value for money in improving the health outcomes of older patients. Furthermore, the study compared two different tools for measuring HRQoL and found that there was no significant difference in their effectiveness in evaluating various interventions. This reinforces the importance of using appropriate measurement tools to accurately assess the cost-effectiveness of healthcare interventions.

Finally, the findings of this study emphasize the need to consider both the clinical and economic outcomes of healthcare interventions on CKD patients, when making decisions about resource allocation. The study's results provide valuable insights for policymakers, healthcare professionals, and other stakeholders in making informed decisions about allocating resources to maximize the health outcomes of older patients with CKD.

## Data Availability

The datasets used and/or analyzed during the current study are available from the corresponding author on reasonable request based on the PERSIAN cohort study protocol electronic requests to the director of the study [https://persiancohort.com/].

## Footnotes

1 It is worth noting that the mapping function used to convert ICECAP-O scores to utility scores may vary depending on the population being studied and the preferences of the individuals involved. Therefore, it is important to carefully select a mapping function that is appropriate for the specific population and context.
